# The Hedgehog Signalling Pathway in Cell Migration and Guidance: What We Have Learned from *Drosophila melanogaster*

**DOI:** 10.3390/cancers7040873

**Published:** 2015-10-02

**Authors:** Sofia J. Araújo

**Affiliations:** Institut de Biologia Molecular de Barcelona (IBMB-CSIC), Parc Cientific de Barcelona, C. Baldiri Reixac 10, 08028 Barcelona, Spain; sarbmc@ibmb.csic.es; Tel.: +34-93-403-4966; Fax: +34-93-403-4979

**Keywords:** hedgehog, patched, *Drosophila melanogaster*, cell migration, guidance

## Abstract

Cell migration and guidance are complex processes required for morphogenesis, the formation of tumor metastases, and the progression of human cancer. During migration, guidance molecules induce cell directionality and movement through complex intracellular mechanisms. Expression of these molecules has to be tightly regulated and their signals properly interpreted by the receiving cells so as to ensure correct navigation. This molecular control is fundamental for both normal morphogenesis and human disease. The Hedgehog (Hh) signaling pathway is evolutionarily conserved and known to be crucial for normal cellular growth and differentiation throughout the animal kingdom. The relevance of Hh signaling for human disease is emphasized by its activation in many cancers. Here, I review the current knowledge regarding the involvement of the Hh pathway in cell migration and guidance during *Drosophila* development and discuss its implications for human cancer origin and progression.

## 1. Introduction

The Hedgehog (Hh) signaling pathway is involved in a plethora of cellular processes including cell proliferation, patterning, axonal guidance, and angiogenesis. A surprising number of apparently unrelated human diseases, including several cancers and a number of syndromes and malformations, seem to be associated with the abnormal function of the Hh pathway. Initial studies of Hh signaling were mainly built on the genetic analysis of development in the fruitfly *Drosophila melanogaster*. Yet, homologues were later found in many species where they were also shown to play essential roles in development and disease [1]. Nevertheless, the extent to which Hh signaling is generally required for cell migration and guidance during *Drosophila* and/or vertebrate development is still matter of debate, despite the burst of activity in this field in recent years [2,3]. The effects of Hh as a morphogen make its involvement in cell behavior difficult to ascertain and, thus, genetically-amenable models are needed for the study of the complete implications of Hh signaling in cell migration and guidance. Over the years, *Drosophila* has been on the forefront on Hh research and I will focus on the involvement of the Hh pathway in regulating cell migration and guidance in this model organism.

Hh was first identified in *Drosophila* as a secreted protein that directs pattern formation and can act as a short-range, contact-dependent factor, as well a long-range, diffusible morphogen [4,5]. There are four key players in canonical Hh signaling: Hh, its membrane receptor Patched (Ptc), the G-protein-coupled receptor Smoothened (Smo), and the transcription factor Cubitus interruptus (Ci). During normal Hh signaling, binding of Hh to its receptor will relieve Ptc-dependent repression of Smo and activate downstream signaling events that include a cascade of protein interactions, post-translational modifications, and proteolysis events. In the absence of Smo activity, Ci is phosphorylated and targeted for processing into a shorter cytoplasmic repressor form. Activation of Smo, which also involves phosphorylation, inhibits Ci phosphorylation causing the accumulation of full length Ci, which can translocate into the nucleus and activate gene expression [3,6]. Transcriptional downstream targets of canonical Hh signaling include the up-regulation of its own receptor Ptc, signalling molecules like Decapentaplegic (Dpp), other transcription factors such as Engrailed (En) and cell cycle regulators like Cyclin D (CycD) [7]. Apart from binding Hh to initiate downstream signaling events, Ptc also modulates the extracellular gradient of Hh in a way that is functionally distinct from its ability to regulate Hh signal transduction [8,9]. Tight control of Hh signaling is vital to maintain proper gene expression during cell growth, survival, and differentiation in a variety of tissues [3,10]. Consequently, up-regulation of Hh signaling has been linked to several types of cancer [11]. Remarkably, despite the importance of identifying Hh target genes, little is still currently known about the genes whose expression is controlled, directly or indirectly, by Hh activity and the cellular consequences of this signaling activation.

Cell migration is a widespread and complex cellular activity crucial to many biological processes such as embryonic development and invasion and metastasis of human cancers. As individual and groups of cells migrate through complex cellular environments, they are steered toward their target sites by guidance molecules and stop at the sites where their biological function is needed. In contrast to morphogens, which act mainly by inducing transcriptional changes, these guidance molecules act primarily by regulating cytoskeletal and membrane dynamics. Clearly, expression modulation and molecular localization of these guidance molecules is very important for both morphogenesis and human disease.

In *Drosophila* embryos, well-studied models of cell migration include the border cells, the germ cells, and the tracheal and nervous system cells. The Hh signaling pathway has been reported to be involved in cell migration in all of these model systems.

## 2. Hh Signaling in Border Cell Migration

One key model to cell migration is the stereotyped movement of border cell clusters in the *Drosophila* ovary. The *Drosophila* ovary is composed of progressively developing egg chambers arranged in a linear fashion, each of which contains 15 nurse cells and one single oocyte surrounded by somatic follicle cells. One pair of specialized follicle cells, the polar cells, differentiates at each end of the egg chamber. At the anterior end, the border cell cluster is formed by the polar cells and several neighboring follicle cells. Over many hours during ovarian development, border cells migrate as a unit between nurse cells toward the oocyte. The specification and directionality of border cell movement are regulated by hormonal and signaling mechanisms [12]. Border cells have a stereotypical migration pattern that is carefully regulated by the action of at least four well-known signaling pathways. Specification of border cell identity and regulation of border cell motility is achieved by the JAK/STAT pathway [13,14,15]. Migration timing is coordinated by steroid hormone signaling through the Ecydsone receptor (EcR) [16]. In order to maintain the cluster as a cohesive unit throughout migration, proper levels of cell adhesion between border cells are promoted by JNK signaling [17]. Finally, to direct border cells to the oocyte, growth factors secreted by the oocyte activate Platelet Derived Growth Factor receptor/Vascular Endothelial Growth Factor receptor (PDGFR/VEGFR) and epidermal growth factor receptor (EGFR), two receptor tyrosine kinases (RTKs) expressed on the border cells [18,19].

As a morphogen, Hh promotes follicle cell differentiation during *Drosophila* oogenesis [20], and recently two independent genetic screens have identified the involvement of Hh in border cell migration [10]. In this study, authors have found that interference with Hh signaling by knockdown resulted in incomplete cell migration, pointing at a positive effect of Hh signaling in cell movement. More specifically, they found an interaction between Hh signaling and both Rac and the polarity protein Par-1 that mediates proper migration and propose that Hh may be required to maintain the subcellular localization of E-cadherin to facilitate cell movement [10]. Based on their results, the authors speculate that the Hh involvement in border cell migration is not as a long-range chemoattractant, but as an inducer of cell shape changes.

## 3. Hh Signaling in Germ Cell Migration

In most organisms, germ cells are set aside as a special population early in development. In *Drosophila*, primordial germ cells (PGCs) are the first to individualize from the syncytial embryo. Early in development, the PGCs are formed at the posterior pole through the action of maternally derived cytoplasm components such as Oskar (Osk), Vasa (Vas), and Tudor (Tud) [21]. Since PGCs and the somatic cells of the gonad originate in disparate locations, the germ cells must undergo a stereotyped migration to find and associate with the somatic gonad. At the time of gastrulation, the PGCs adhere to the underlying somatic cells and become internalized, localizing inside the posterior midgut pocket. Next, the germ cells begin actively migrating across the epithelium of the posterior midgut [22]. Finally, PGCs migrate away from the midgut toward the adjacent mesoderm where they associate with the somatic gonadal precursors (SGPs) [23]. Once associated with the SGP clusters, the germ cells round-up, coalesce, and form a gonad on each side of the embryo. Germ cell migration is regulated by the enzyme 3-hydroxy-3-methyl-glutaryl coenzyme A reductase (Hmgcr). In *hmgcr* mutant embryos many germ cells fail to reach the SGPs leading germ cells to “scatter” in the soma by the time the SGPs and germ cells coalesce [24]. *hmgcr* is highly expressed in the SGPs where it is crucial for PGC attraction to SGPs. As the ultimate proof of its capacity for chemoattraction, ectopic expression of *hmgcr* leads to attraction of germ cells to the ectopic tissue [24]. Thus, *hmgcr* expression leads to an attractive cue for germ cells that guides their migration.

Several studies have suggested that Hh is involved in cell guidance during PGC migration [25,26] and that Hh is downstream of the Hmgcr pathway [27,28]. It is reported in these studies that Hh is expressed in the gonadal mesoderm and that ectopic expression of Hh leads to germ cell migration defects [25]. Also, germ cells compromised for Ptc function clump prematurely, whereas *smo* germ cells scatter randomly [25]. Based on these studies, the authors propose that Hh acts as a diffusible chemoattractant that guides *Drosophila* germs cells to the somatic gonad [28]. In addition, Deshpande and coworkers have found that Hmgcr promotes Hh signaling and that genes involved in Hh transmission, such as *tout-velu* (*ttv*) and *shifted* (*sft*), are also required for PGC migration [26,29]. However, there is still a certain controversy regarding Hh involvement in PGC migration. Evidence from genetic screens for mutants that affect germ cell migration have failed to identify a role for components of the Hh signaling pathway [30]. Furthermore, Renault and coworkers have reported that the ability of Hmgcr to attract germ cells does not act through Hh [30]. Further studies are clearly needed to establish a connection between Hh signaling to the germ cells, cytoskeletal changes and directed movement.

## 4. Hh Signalling in Tracheal Cell Migration

The tracheal system of *Drosophila* is a branched epithelial tubular network that delivers oxygen to all tissues. It is an invertebrate model for tubulogenesis, vasculogenesis, and angiogenesis and a model of choice for learning how extracellular signals transduce into collective cellular movement [31]. The tracheal system of *Drosophila* arises from the tracheal placodes, clusters of ectodermal cells that differentiate at each side of 10 of the embryonic segments (T2 to A8). The cells of each cluster invaginate and migrate in a stereotypic pattern into a complex array, forming a network of multicellular tubes. Tracheal cell migratory directionality relies on a set of positional cues given by the surrounding cells in the embryo, which is based in ligand-receptor contacts [32,33,34,35,36,37]. In addition, tracheal cells interact directly with their underlying substrates, a process ultimately determined by molecules expressed at their surface [38,39,40,41].

Throughout the entire migratory process, tracheal cells do not divide and remain polarized along the apical-basal axis. During tracheal cell migration, the main chemoattractant is the Fibroblast Growth Factor (FGF) homologue Branchless (Bnl) [32]. Bnl chemoattraction is achieved by its activation of the FGF receptor (FGFR) Breathless (Btl) on tracheal tip-cells [42,43]. *bnl* is expressed in a complex and dynamic pattern in tissues surrounding the developing tracheal system and works at the top of a hierarchy of cell interactions that orchestrate the branching process [32]. Both the earliest branching events and the later programs of tracheal gene expression are determined by the proper spatio-temporal expression of *bnl*. Ectopic expression of *bnl*, in the CNS for example, leads to high numbers of tracheal cells migrating towards this tissue [44]. Excess of *bnl* expression leads to extra tracheal migration and branching, but it is also known that the absence, as well as an excess of *bnl* expression, can inhibit cell migration [32,44,45].

Hh is expressed segmentally in ectodermal stripes and plays an active role in tracheal development from early stages. In *hh* mutants, already by stage 11 of embryonic development, tracheal defects are detectable. Each placode loses six to eight cells, which remain on the ectoderm after invagination [46]. Additionally, in *ptc* mutant embryos, less cells account for the tracheal tree at later stages independently of apoptosis [45]. This suggests that both high and low levels of Hh signaling affect tracheal cell fates, despite *trachealess* (*trh*) expression not being dependent on *hh* [7]. In a further effort to clarify the effects of Hh in tracheal cell fates, this morphogen has been recently shown to be involved in branching pattern and tube shape diversity together with WNT/Wingless signals [47].

Remarkably, the lack of cell migration is the most severe tracheal defect observed in *hh* mutant embryos from embryonic stage 12. The tracheal tree is stalled, with most of the cells remaining at the level of the placode region, and forming only a kind of transverse connective branch [46]. Glazer and Shilo also reported that compromising the activity of the Hh pathway reduced the extent of terminal branching, while its activation gave rise to an excess of terminal cells in the dorsal branch [46]. Together with Decapentaplegic (Dpp), Hh was also shown to act on terminal cells during their cytoplasmic extension and elongation, exerting its attractive effect from the epidermis [48].

**Figure 1:**
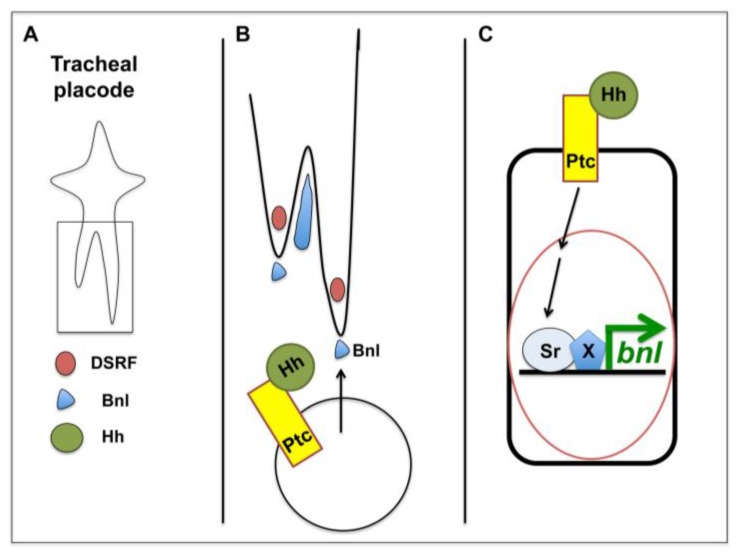
Hh in tracheal cell migration. Schematic representation of one tracheal placode at stage 13; (**B**) detail of (**A**), showing the tip cells of each branch and how Bnl is expressed in the surrounding tissues in order to provide a chemoattractive signal; and (**C**) how canonical Hh signaling induces the expression of Bnl in the cells surrounding the tracheal tip cells (adapted from [45]).

The involvement of Hh in tracheal cell migration has been attributed to a direct effect in the extension of the terminal cells of the dorsal branch [48]. An indirect role through the positive regulation of *bnl* expression has also been reported [45]. In addition, *bnl* expression was shown to be affected in *hh* mutants and *hh* mutant tracheal trees resemble the ones where *bnl* is down-regulated [46]. So, by regulating *bnl* expression, Hh is able to modulate cell migration non-autonomously (Figure 1). Furthermore, the Hh signaling pathway regulates *bnl* expression via Stripe (Sr), a transcription factor with homology to the Early Growth Response (EGR) family of vertebrate transcription factors [45]. It is likely that most of the migratory defects seen in *hh* mutants, are due to *bnl* expression changes. Interestingly, an over-activation of Hh signaling as seen in *ptc* mutants and conditions where the canonical Hh pathway is activated, also induce migration defects [45]. This is in accordance with previous findings where fine-tuning of spatio-temporal expression of *bnl* is crucial for the correct migration of tracheal cells.

## 5. Hh signaling in Axonal Guidance

During axonal guidance, the principles underlying growth cone motility and guidance are similar to those guiding cell migration. A well-known model system to study axonal guidance is the ventral nerve cord (VNC) of *Drosophila*, which has proven to be a useful model for understanding both neurogenesis and axon guidance [49]. During axonal guidance the growth cone travels over long distances, and both its formation and consequent advancement are tightly regulated by extracellular factors and intracellular signaling molecules. In *Drosophila*, the array of axons composing the CNS has a ladder-like structure: each body segment comprises an anterior and a posterior commissural tract that cross the midline and join one of the two lateral longitudinal tracts that extend the length of the embryo. During development, commissural neurons send axons that project toward and, subsequently, across the midline, which acts as an intermediate target and influences axonal trajectories by expressing attractive and repulsive cues [50]. Over the years, substantial progress has been made in identifying the ligands and receptors that define the path taken by individual axons, both in vertebrates and invertebrates [49,51]. Ligand/receptor pairs include members of the Netrin/Deleted in Colorectal Cancer (DCC), Slit/Robo, and Semaphorin/Plexin/Neuropilin families. More recently, the repertoire of potential guidance factors has been expanded to include morphogens, signaling molecules that originate from a restricted region of a tissue and spread away from their source to form concentration gradients that can span many cell diameters. This has added complexity to the repertoire of wiring instructions for navigating growth cones. These morphogens, which tend to set up gradients along major body axes, remain or become re-expressed in the region, to provide directional information to axons along major body axes. To date, some morphogens have been shown to have important and evolutionary conserved roles in axonal guidance in vertebrates: these comprise members of the Bone Morphogenetic Protein (BMP), Hedgehog (Hh), Wnt and Fibroblast Growth Factor (FGF) families [52,53]. In *Drosophila*, it has been shown that Wnt5 acts directly in axon guidance through Derailed (Drl) [54]. At later developmental stages, morphogens are important in shaping topographic projections in both the chick and the fruitfly visual systems [55]. However, until recently, it had not been properly established if Hh had a direct role in axonal movement in *Drosophila*.

Hh has been reported to regulate the expression of many genes in the *Drosophila* nervous system. Both *hh* and *ptc* mutant embryos display very strong phenotypes in the Central Nervous System (CNS). In *hh* mutants, many midline cells die and surviving midline cells are undifferentiated [56]. More specifically, Hh represses anterior midline glia (AMG) and activates posterior midline glia (PMG) gene expression and determines neuronal fate in the posterior of each segment [57,58]. In addition, in *ptc* mutant embryos very few commissures are properly formed and most of these defects have been attributed to mis-specification and neuronal loss [59,60]. However, the axonal defects observed in *hh* and *ptc* mutants may be attributed also to alterations in chemoattraction and/or chemorepulsion and not just to changes in cell fate.

**Figure 2:**
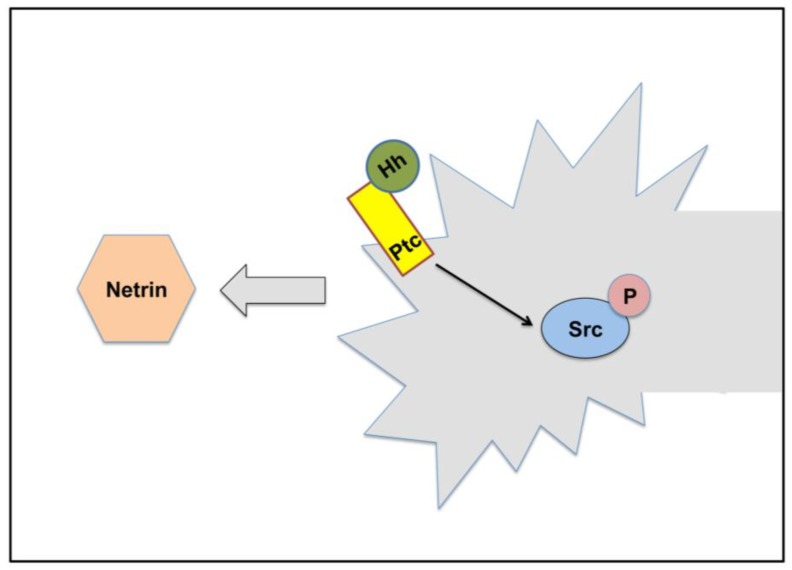
Hh signaling in axonal guidance in *Drosophila*. Schematic representation of a growth cone and how Hh signaling affects its response to Netrins. Non-canonical Hh signaling induces Src phosphorylation, cooperating with Netrin/Fra (DCC) axonal attraction.

Recently, it has been shown that Hh is involved in axonal guidance during embryonic stages, acting in midline axonal guidance together with the Frazzled (Fra)/DCC axonal guidance receptor (Figure 2). In addition, through genetic experiments Ricolo and coworkers were able to show that the involvement of Hh in axonal guidance is most likely accomplished via a non-canonical Hh signaling pathway [61]. So, as in vertebrates, Hh is also involved in embryonic axonal guidance in *Drosophila*.

## 6. Hh signaling in Glial Cell Migration

Glial cells provide a variety of cues for growth cone guidance and are critical determinants of neuronal connectivity. In many cases glial cells migrate over long distances before reaching their final positions. During embryogenesis, peripheral glia migration is in part controlled by Netrin signaling and the modulation of adhesive forces is provided by the expression of the *Drosophila* NCAM homolog Fasciclin2 (Fas2) [62,63,64]. CNS glia rely on the presence of the neuronal substrate to initiate and direct their migration [65,66]. Some aspects of glial migration are regulated by the Slit/Roundabout ligand/receptor system that controls axonal guidance across the CNS midline [67]. In the *Drosophila* larva, the developing visual system provides an easily accessible model system with which to study glial migration. Photoreceptor neurons emerge in the eye imaginal disc (eye disc), which is connected to the brain by the optic stalk. All retinal glial cells originate from the optic stalk glia and subsequently migrate to the eye disc [68,69]. A signal released from developing photoreceptor cells in the eye disc guides these glial cells [69].

In the eye-disc, loss-of-function *hh* mutants exhibit precocious glial cell migration though glial cells do not require the canonical Hh pathway to migrate [70]. Part of the signaling mechanism is conveyed by Hh and the casein kinase Iγ Gilgamesh (Gish). In midline glial guidance, *fear-of-intimacy* (*foi*) acts non-cell autonomously to terminate glial cell migration and genetic evidence suggests that *foi* interacts with the Hh pathway [71]. Interestingly, loss-of-function of *foi* affects the guidance of not only glial cells but also germ cells, somatic muscle and tracheal cells [71,72]. The only genetic evidence for an interaction between *foi* and *hh* comes from Pielage and coworkers, however, it would be very interesting to ascertain if *foi* is one of the missing links between cell migration in these systems and the Hh pathway.

## 7. Same Signaling Ligand for Different Cellular Responses

Hh and its canonical and non-canonical downstream targets tightly regulate key cellular behaviors, such as proliferation, differentiation, migration, and survival both in vertebrates and invertebrates [2,5]. A great deal of knowledge has been generated by studies of this morphogen in *Drosophila melanogaster*. Regarding cell migration and guidance, we have learned that Hh can act as a direct or indirect chemoattractant or by regulating cellular physiology, both in a canonical and in a non-canonical signaling mode (Table 1).

**Table 1:** **Hh as a guidance molecule**. Summary of the Hh effects in cell movement in the various cell migration and guidance models in *Drosophila melanogaster*.

Cell Type	Hh Effect	Hh Signalling	Reference
**Border cells**	Cell-shape changes	Canonical	[10]
**Germ cells**	Chemoattraction	Non-canonical	[25,29]
**Tracheal cells**	Chemoattraction (via Bnl)	Canonical	[45]
**Axons**	Chemoattraction	Non-canonical	[61]
**Glial cells**	Spatio-temporal control	Non-canonical	[70,71]

Remarkably, Hh signaling can act in distinct ways to regulate cellular movement. Thus, integration of all these different models and modes of action, is particularly important to elucidate how this morphogen is involved in so many different responses during development. In addition, it is crucial to differentiate autonomous from non-autonomous effects of Hh signaling in order to get a clear view of its effects in cell migration and guidance. From the examples presented above, we can conclude that sometimes Hh can act as a “typical” chemoattractant, inducing cellular changes such as the modulation of E-cad in border cells, whereas in others it acts more as a morphogen, leading to the transcription of other ligands such as Bnl in the tracheal system.

Many questions remain as to how Hh may participate in cell movement. For instance, is Hh non-canonical signaling involved in Src phosphorylation in germ cell and migration and axonal guidance? Preliminary results in *Drosophila*, seem to indicate that Hh signaling changes pSrc levels in the midline during axonal guidance [61]. Or, is Hh regulation of *bnl* levels also involved in germ cell migration? It is possible to envisage, due to their spatio-temporal developmental characteristics that tracheal cells and germ cells may be influenced by some of the same cues as they migrate. This being the case, one can speculate that there could be an autonomous Hh effect inducing germ cell-shape changes and a non-autonomous one modulating migration. This effect would most likely happen via Bnl-signaling in other tissues, since Btl has not been detected in germ cells. Furthermore, a “cell-shape change” effect similar to the one happening in border cells might be taking place in the tracheal cells themselves. Perhaps direct Hh signaling also induces changes in cell-shape in tracheal cells, which may indeed happen because *ptc* is expressed by tracheal cells [45,46,48]. To add more complexity to the possible scenarios, it has been reported that Bnl regulates glial migration spatio-temporally, activating Btl in the glia, and that these functions are Hh dependent [73]. This may implicate the “Hh-modulating-Bnl-hypothesis” into glial cell migration. And last but not least, *btl* is expressed in border cells and *btl* mutations have been reported to induce border cell migration defects [74]. Cell migration, cell-shape changes and spatio-temporal control of cell movement are processes that when impaired can be on the onset and progression of human cancer. Due to the implication of the Hh signaling pathway in human cancers, it is of great importance to elucidate the effects of this morphogen in cell migration and guidance. In order to be able to answer all the remaining questions and to test all hypothesis and further advance on how Hh influences cell migration and guidance, more studies in *Drosophila* and other model organisms are necessary.
